# Case Report: Multiple prolactinomas in a young man with Kallmann syndrome and familial hypocalciuric hypercalcemia

**DOI:** 10.3389/fendo.2023.1248231

**Published:** 2023-10-30

**Authors:** Mojca Jensterle, Andrej Janež, Tina Vipotnik Vesnaver, Maruša Debeljak, Nika Breznik, Katarina Trebušak Podkrajšek, Rok Herman, Eric Fliers, Tadej Battelino, Magdalena Avbelj Stefanija

**Affiliations:** ^1^ Department of Endocrinology, Diabetes and Metabolic Diseases, University Medical Centre Ljubljana, Ljubljana, Slovenia; ^2^ Faculty of Medicine, University of Ljubljana, Ljubljana, Slovenia; ^3^ Clinical Institute of Radiology, University Medical Centre Ljubljana, Ljubljana, Slovenia; ^4^ Clinical Institute for Special Laboratory Diagnostics, University Children’s Hospital, University Medical Centre Ljubljana, Ljubljana, Slovenia; ^5^ Institute of Biochemistry and Molecular Genetics, Medical Faculty, University of Ljubljana, Ljubljana, Slovenia; ^6^ Department of Endocrinology and Metabolism, Amsterdam Gastroenterology, Endocrinology and Metabolism, Amsterdam UMC, University of Amsterdam, Amsterdam, Netherlands; ^7^ Department of Paediatric Endocrinology, Diabetes and Metabolic Diseases, University Children’s Hospital, University Medical Centre Ljubljana, Ljubljana, Slovenia

**Keywords:** Kallmann syndrome, prolactinoma, hypogonadism, sex hormones, familial hypocalciuric hypercalcemia

## Abstract

**Introduction:**

The occurrence of prolactinomas in sex hormone treated patients with central hypogonadism is extremely rare.

**Case presentation:**

We present a Caucasian male patient who was diagnosed with Kallmann syndrome (KS) at age 15 years. Testosterone treatment was started. At age 26 the patient presented with mild headache. MRI revealed two separate pituitary adenomas along with the absence of the olfactory bulbs. Given the presence of marked hyperprolactinemia (17x upper limit of the reference range) the diagnosis prolactinoma was made and treatment with cabergoline was started which resulted in a complete biochemical response and in marked reduction of both adenomas in size. Hypogonadism persisted and testosterone replacement therapy was continued. Genetic testing of genes associated with pituitary tumors, Kallmann syndrome and idiopathic hypogonadotropic hypogonadism was negative. Mild concomitant hypercalcemia in accordance with familial hypocalciuric hypercalcemia (FHH) prompted mutation analysis of the calcium receptor (*CASR)* gene which yielded a pathogenic inactivating variant.

**Discussion/conclusion:**

The presence of two separate prolactinomas in a patient with KS has not yet been reported in the literature. The effect of sex hormone treatment of KS patients on the possible development of prolactinoma is unknown at present. The occurance of multiple prolactinomas in our patient suggests increased susceptibility. Although CaSR is expressed in GnRH neurons in mouse brain and CaSR deficient mice have a reduced hypothalamic GnRH neuronal population, the relevance of the *CASR* gene variant in our patient for the KS phenotype is unclear at present.

## 1 Introduction

Prolactinomas are the commonest pituitary adenomas, representing about 50% of pituitary adenomas, with an overall prevalence of approximately 50:100,000 (1, 2). The vast majority of pediatric prolactinomas are identified in late adolescence (2), while they are ultra-rare in prepubertal children (3–5). There is a strong female preponderance, with a peak incidence in childbearing ages (1). In males, peak incidence is beyond 50 years; however, tumors in males are on average larger and more aggressive (1). The sex differences in presentation and biological behavior are associated with variability in expression of genes involved in the estrogen signaling pathway (6). Therefore, hormonal homeostasis was proposed to play an important role in prolactinoma pathogenesis (3).

Kallmann syndrome (KS) is a congenital form of hypogonadotropic hypogonadism (HH) associated with hyposmia/anosmia, that occurs with an incidence of 1:48,000 (1:30,000 males) (7). Genetic variants in various genes underlying KS are associated with embryonal development and migration of GnRH neurons along with the development of olfactory neurons (8). A clinical study in a large group of KS patients showed that certain clinical features are highly associated with specific genetic causes, such that synkinesia (*ANOS1*), dental agenesis (*FGF8/FGFR1*), digital bony abnormalities (*FGF8/FGFR1*), and hearing loss (*CHD7*) can be useful for prioritizing genetic screening (9).

We present a young male patient with a rare co-occurrence of KS and multiple prolactinomas. Genetic screening for KS and for pituitary adenoma was negative. As the patient showed concomitant mild hypercalcemia in accordance with familial hypocalciuric hypercalcemia (FHH) we performed additional mutation analysis of the calcium receptor (*CASR*) gene which yielded a pathogenic inactivating variant. We are unaware of earlier reports of the combined occurrence of these rare clinical features in KS patients.

## 2 Case presentation

A 15-year-old male Caucasian patient presented with absent puberty, small testicles (1 ml), osteopenia and anosmia. The flaccid penile length was 3.6 cm and the lenght of the stretched penis was 5.2 cm. No cryptorchidism or microphallus were reported at birth. Endocrine assessment, including a gonadotropin-releasing hormone (GnRH) stimulation test (serum luteinizing hormone (LH) and follicule-stimulating hormone (FSH) response upon stimulation with gonadorelin 100 µg intravenously, measured at 0’, 20’, 30’ and 60’ min), confirmed HH [basal LH 0.1 U/L (reference range in 11-15 yo boys 0.2-1.9 U/L (10)], peak LH 3.19 U/L [reference range in 11-15 yo boys 1.8-12 U/L (10)], basal FSH 0.24 U/L [reference range in 11-15 yo boys 0.3-3.5 U/L (10)], peak FSH 2.33 U/L [reference range in 11-15 yo boys 1.2-5.5 U/L (10)], serum testosterone 0.5 nmol/L (reference range in 14.5-17.3 yo boys 0.85-45.62 nmol/L) (11). Prolactin was not determined at the time of diagnosis until just before testosterone replacement was started at the age of 15 years old [10.2 mcg/L, upper limit of the reference range 16.1 mcg/L (12)]. Based on concomitant olfactory dysfunction, he was diagnosed with KS. No baseline brain imaging was performed at that time. Hormonal replacement treatment was initiated for puberty induction with testosterone enanthate and then continued with testosterone undecanoate following the local monitoring protocol. Testosterone levels were sustained within the normal range and bone mineral density reached normal adult levels by the age of 21.5 years (femur neck Z-score -0.5 SD and lumbar spine Z-score -0.8 SD).

**Figure 1:**
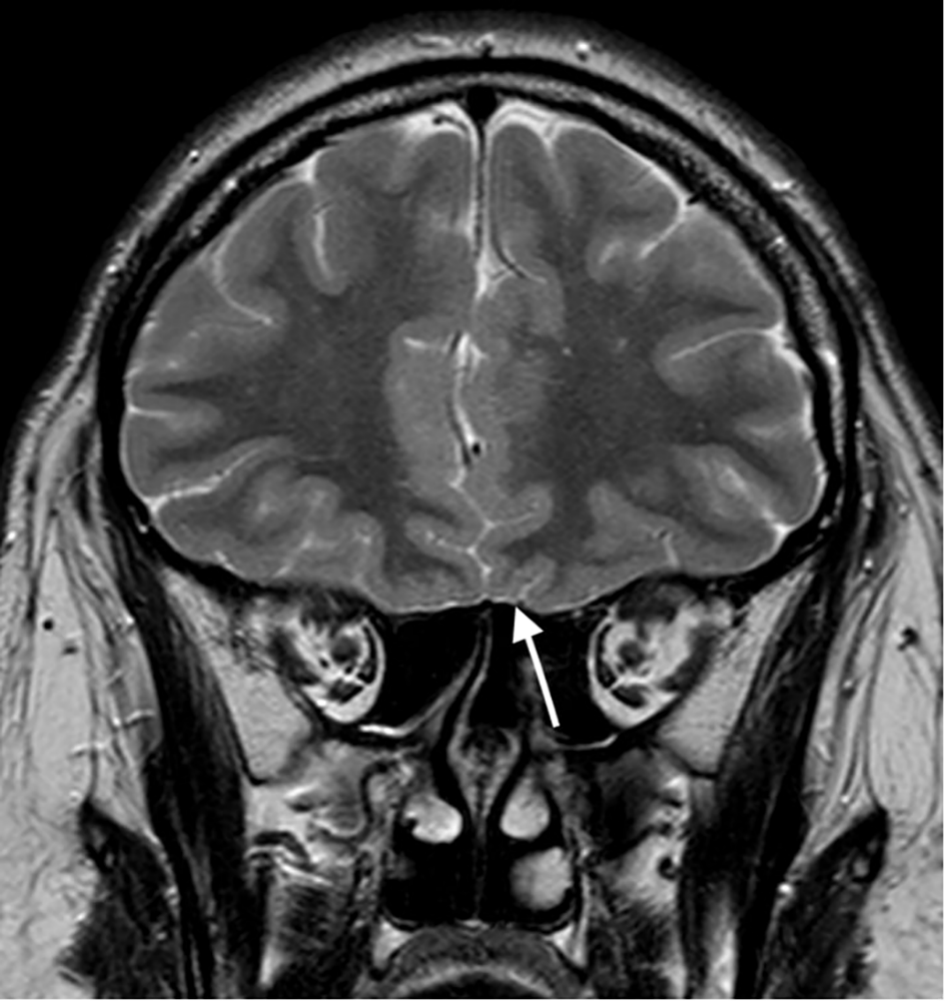
T2 weighted image in coronal plane; Absent olfactory bulbs (arrow).

**Figure 2:**
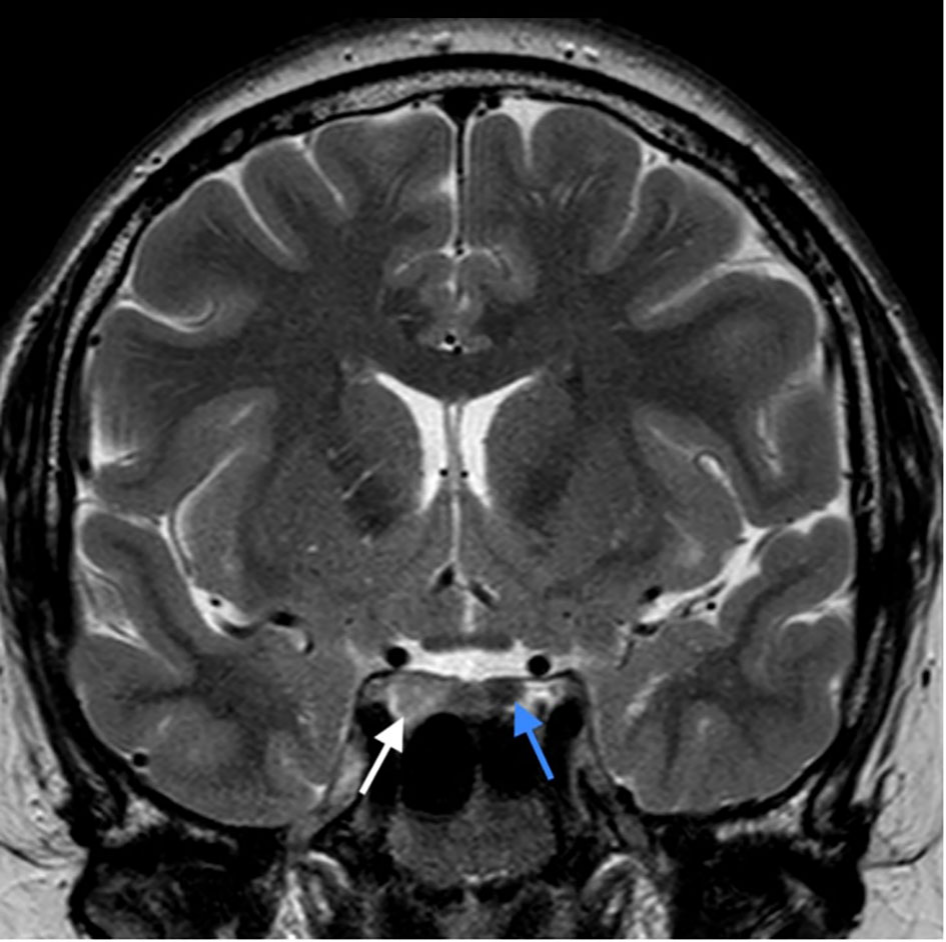
T2 weighted image in coronal plane; pituitary adenoma of the right lobe of the gland, extending to the right parasellar region (white arrow), rounded adenoma of the left lobe (blue arrow). Pituitary infundibulum is slightly tilted to the left.

**Figure 3:**
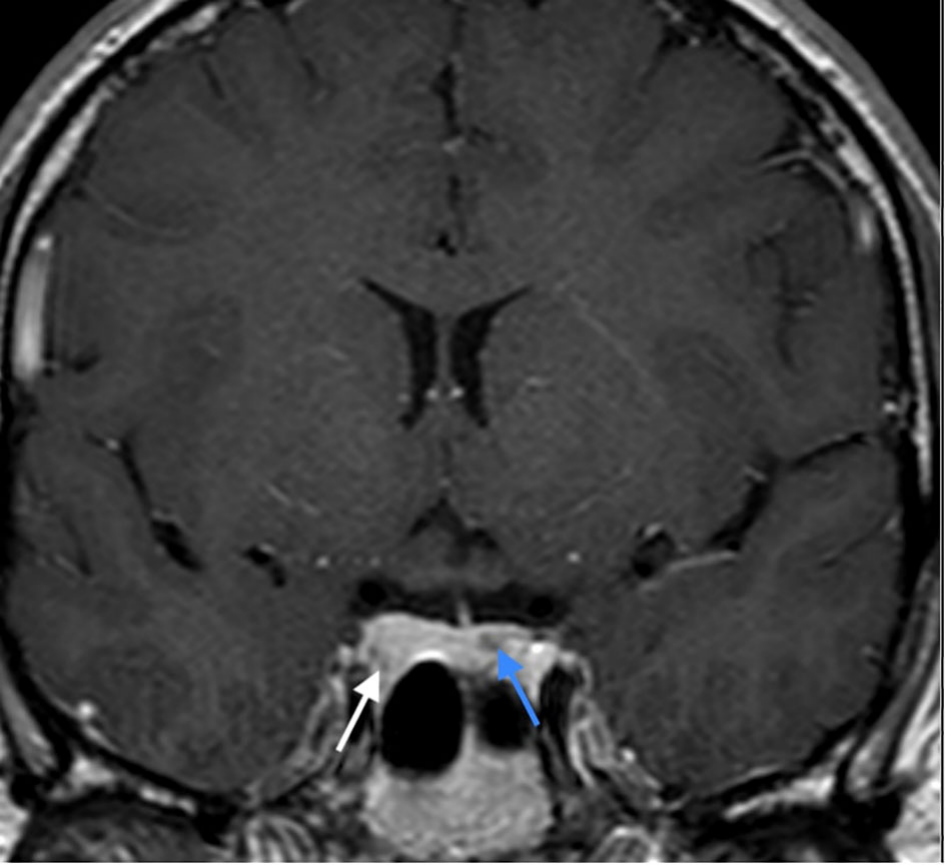
CE T1 weighted image in coronal plane: enhancement of the lesion in the right lobe (white arrow), lesser enhancement of the lesion in the left lobe (blue arrow).

At age 26 the patient presented with mild headache. MRI brain imaging (1,5T Philips Achieva MRI scanner) revealed the absence of olfactory bulbs (shown in **Figure 1**) (13). Unexpectedly, there were two additional abnormal findings in the pituitary region, i.e., a T1 hypointense, T2 hyperintense lesion, probably adenoma measuring 10 x 8 mm that enhanced after Gadolinium enhancement. The lesion expanded the right lobe of the pituitary gland and extended to the right parasellar region. Additionally, a second lesion was present in the left lobe of the gland, measuring 5 mm in diameter. The second adenoma was slightly hyperintense on T1 sequence, T2 hypointense, and after gadolinium it enhanced less than the rest of the gland (shown in **Figures 2**, **3**).

**Figure 4:**
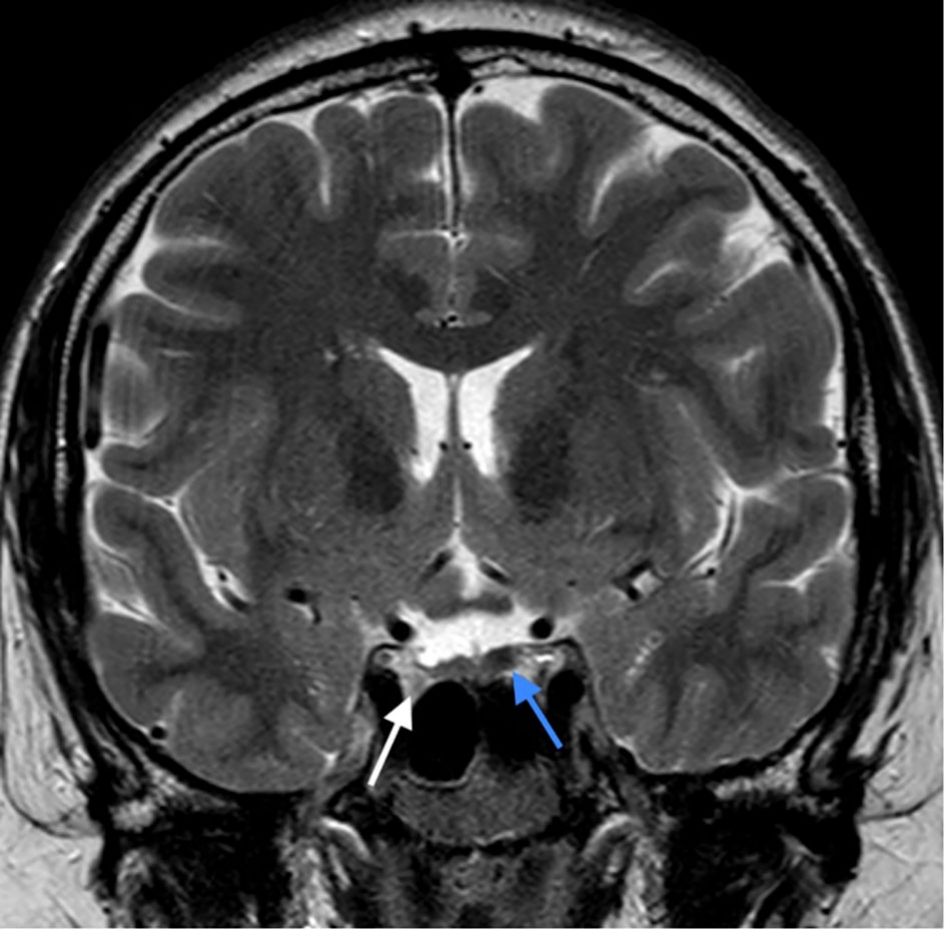
The latest MRI assessment 3 years after initiating the cabergoline treatment: Control T2 weighted image in coronal plane: Size reduction of the right lobe adenoma (white arrow) and almost complete disappearance of the lesion in the left lobe (blue arrow).

**Figure 5:**
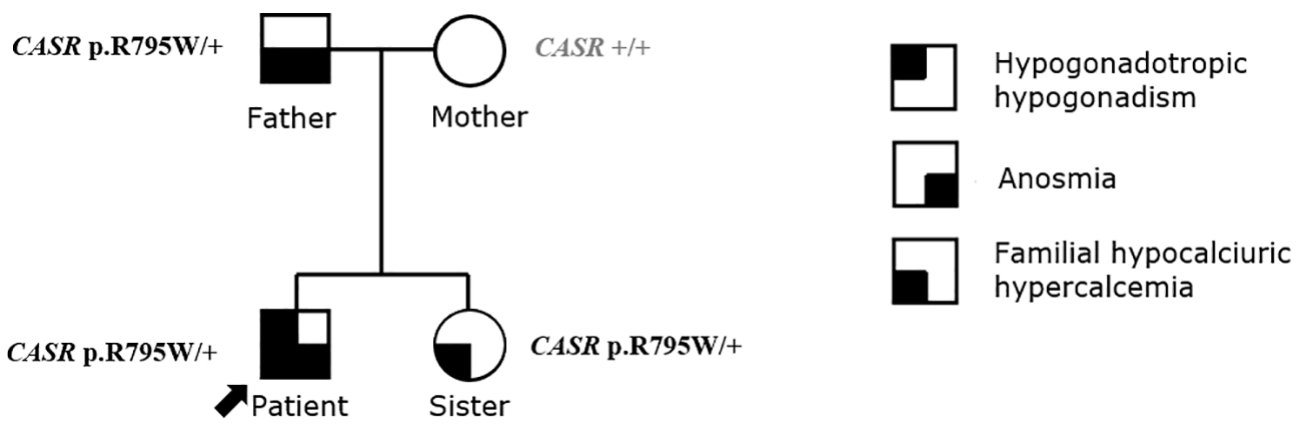
The pedigree and the diagnosis of family history.

Hormonal evaluation revealed hyperprolactinemia (prolactin 292 mcg/L, upper limit of the reference range for men 17 mcg/l). Deficiencies of thyrotropin (TSH), adrenocorticotropin (ACTH), and growth hormone (GH) as well as GH hypersecretion were excluded. TSH was 3.95 mE/L (reference range 0.55-4.78 mE/L), fT4 was 14.5 pmol/L (reference range 11.5-22.7 pmol/L), ACTH was 5.46 pmol/L (reference range up to 10.2 pmol/), basal cortisol was 406 nmol/l, dehydroepiandrosterone (DHEAS) was 11.10 µmol/L (reference range 7.56-17.28 µmol/L), insulin-like growth factor 1 (IGF-1) was 223 mcg/L (reference range 75-274 mcg/L), insulin-like growth factor-binding protein 3 (IGFBP-3) was 4.69 mg/L (reference range 2.80-6.30 mg/L). Treatment with cabergoline at an initial dosage of 0.25 mg twice weekly up-titrated to 0.5 mg twice weekly resulted in a complete biochemical response (prolactin level 23.9 mcg/L after 3 months, 8.1 mcg/L after 6 months and 2.2 mcg/L at the last check-up 3.5 years after starting cabergoline). Both pituitary lesions showed a clear reduction in size, and the lesion in the left lobe almost completely disappeared (shown in **Figure 4**). The frequencies of mild headaches have decreased, not necessarily related to reduction of the lesions. After normalization of prolactin, we discontinued testosterone undecanoate for 7 months and reassessed the gonadal axis. During that time he continued to take cabergoline. Hypogonadism persisted despite prolactin normalization (testosterone 4.6 nmol/L, reference range 6.9-23.3 nmol/L) and testosterone undecanoate 1000 mg was reintroduced and applied every 12 weeks to sustain testosterone level within the normal range. At last follow up visit, the patient reported no symptoms of hypogonadism, exhibited normal male hair growth pattern and pseudogynecomastia without notable glandular tissue. In addition to the endocrine abnormalities the patient had asymptomatic and mild hypercalcemia (Ca 2.95 mmol/L (ref. range 2.10-2.60 mmol/L) in the presence of elevated plasma PTH (PTH 69 ng/L, ref. range 12-65 ng/L). Morphological examinations of parathyroid glands yielded normal results. A diagnosis of familial hypocalciuric hypercalcemia (FHH) was made based on the combination of these laboratory findings and his family history with a father and sister both known with FHH (shown in **Figure 5**). While other family members had timely puberty (menarche at 13 years in the mother and at 14 years in the sister), his father had anosmia and reportedly shawed his beard regularly at 19 years old. In the extended family there was a history of a throat carcinoma and colon carcinoma in two grandaunts on paternal side.

In all four family members whole genome sequencing using Illumina NovaSeq 6000 System (San Diego, California, USA) was performed. The analysis was focused on exonic and splicing variants. Identified genetic variants with coverage >10x and read frequency >0.3 were annotated and filtered with VarAFT software (14). The minor allele frequency threshold was set at 1% and all variants exceeding this value were excluded from further analysis. Candidate variants were subsequently confirmed with targeted Sanger sequencing. We used ClinSV framework, that enables the identification of copy-number-neutral structural variants and overlapping deletions/duplications events in genome (15). Targeted analysis of 36 genes associated with neuroendocrine tumors (**Supplementary Table 1**), of 63 genes associated with hypogonadotropic hypogonadism (**Supplementary Table 2**) and *TSHZ1* gene associated with isolated congenital anosmia (ICA) did not identify likely pathogenic variants. In an extended panel of 366 candidate genes (**Supplementary Table 3**) based on their biological function in GnRH neuronal development and action (16) and in 191 genes (**Supplementary Table 4**) demonstrated to be downregulated in prolactinoma cells (17) no pathogenic variants according to ACMG criteria (18) were identified. Also, no potentially here described disease related *de novo* variants were identified in the proband. A few variants of unknown significance were identified in genes associated with GnRH neuronal development and action, whose association with KS in our patient is highly unreliable, taking into account lacking additional clinical signs expected in certain gene defects and/or uncertain results of the in silico pathogenicity prediction tools (**Supplementary Table 5**). Familial hypocalciuric hypercalcemia (FHH, OMIM **#** 145980) due to inactivating heterozygous variant in the calcium-sensing receptor (*CASR* NM_000388.4: c.2383C>T, NP_000379.3: p.R795W; a pathogenic variant fulfilling the following ACMG criteria: PM1, PP2, PM2, PP3, PP5) was confirmed align with asymptomatic hypercalcemia and elevated PTH.

## 3 Discussion

We illustrate a patient with Kallmann syndrome and two prolactinomas during early adulthood. Multiple separate pituitary adenomas are generally very rare, identified in only 0.7% of pituitary adenoma cases (19). While increased frequency of unspecified microadenomas have been reported in idiopathic HH patients (20), there is to our knowledge only one anecdotal report of prolactinoma in KS (21). To our knowledge, the occurrence of two separate prolactinomas in a patient with KS has not yet been reported in the literature.

Identification of prolactinomas in our patient first challenged the diagnosis of KS as the cause of hypogonadism, particularly in the absence of genetic confirmation. Only about 50% of idiopathic HH cases are explained by genetic defects (22). We do consider the cause of HH in our patient to be congenital for the following arguments: i) small testicular volume at age 15 indicated long lasting hypogonadism, ii) anosmia with absent olfactory tracts in the patient and iii) anosmia in a family member suggesting an inherited cause. Furthermore, the normal prolactin level just before the start of testosterone replacement strengthens the argument that the patient’s congenital HH was not due to hyperprolactinemia and that there were no prolactin-secreting microprolactinomas at the time of diagnosis. Moreover, hypogonadism was not reversed after adequate prolactin suppression. Longer-term HPG axis suppression despite achieving normoprolactinemia is not unusual in men with macroprolactinomas. According to Sehemby M et al., the likelihood of HPG axis recovery is associated with baseline prolactin level and tumor size. Specifically, tumor diameter less than or equal to 3.2 cm and serum prolactin less than or equal to 2098 ng/mL best predicted reversal of HH (23). Baseline characteristics of our patient were far from reaching these limits.

The only pathogenic variant identified in our patient by targeted phenotype driven analysis was inactivating variant in *CASR* gene, previously described in association with familial hypocalciuric hypercalcemia (FHH) (24). Of note, animal and *in vitro* data shows that CaSR is expressed *in vivo* in GnRH neurons in mouse brain. Moreover, high Ca2+ induces chemotaxis of GnRH cell lines acting via the CaSR, and CaSR deficient mice have markedly reduced GnRH neuronal population in the anterior hypothalamus (25). This points to a possible role for the CaSR pathogenic variant in the development of KS in our patient. On the other hand, no delayed puberty, infertility or central hypogonadism have been reported in FHH patients (26). Thus, at present, the phenotype in our patient cannot be fully explained by the *CASR* variant.

We failed to perform brain or pituitary MRI at baseline in the present patient, and a recent consensus statement supports cranial MRI at baseline in the workup of congenital isolated HH patients, not only to assess inner ear, midline structures and olfactory structures, but also to identify potential tumors and/or space occupying lesions (27). Sellar abnormalities such as craniopharyngioma, intracranial cysts, empty sella, non-functional pituitary adenoma and also prolactinoma have been reported in patients with KS (21, 28–30). On the other hand, the cost-effectiveness of brain imaging in congenital isolated HH has been debated as unsuspected structural lesions of etiological significance were observed in only 1-3% of patients in a larger cohort (31).

Data on the prevalence of structural pituitary abnormalities in men presenting with adult-onset isolated HH is scarce (20, 28, 32–34). A significant ambiguity still remains about which patient deserves a magnetic resonance imaging (MRI) scan of the hypothalamus and pituitary during evaluation and follow-up in this population (28). One study suggests that the use of routine hypothalamic-pituitary imaging in the evaluation of adult-onset isolated HH, in the absence of clinical characteristics of other hormonal loss, hormonal hypersecretion or sellar compression symptoms, does not increase the diagnostic yield of sellar structural abnormalities over that reported in the general population (32). On the other hand, it was reported that structural pituitary disease is more common in adult-onset isolated HH than in the general population, and that current guidelines do not accurately identify ‘at-risk’ individuals (20, 33). A recent report suggested that MRI of the pituitary is not warranted in all patients with adult-onset isolated HH, as the yield of identifiable abnormalities is quite low (34). Anatomic lesions were likely to be present only when low levels of testosterone are found concomitantly with high levels of prolactin and/or low IGF-1 standard deviation score (34). Based on current European Academy of Andrology and Endocrine Society clinical practice guidelines the overall cost-effectiveness of MRI scanning in the absence of clinical evidence of pituitary mass effects is relatively low, but should be considered when testosterone concentrations are <6 nmol/L and LH is low or normal (35, 36).

We identified one case reporting a prolactinoma in a patient with KS (21). The imaging diagnostic in that case was not performed at routine checkup, but only after a life-threatening complication due to pituitary apoplexy (21). By the same authors, an adult-onset prolactinoma in a female with *PROP1* related hypopituitarism including central hypogonadism supplemented with sex hormones was reported (21). The authors speculated that long lasting therapy with sex steroids initiated at adolescence may have facilitated the development of the prolactinoma via epigenetically mediated gene dysregulation (21). Estrogen receptors are commonly present on prolactinoma cells and their density diminishes with dopamine antagonists. It is speculated that the presence of estrogen receptors improves response to dopamine antagonists (37). Of note, aromatase enzyme is expressed in human pituitary in men and women with particularly striking interindividual variance in men (38). As the genetic analysis results for neuroendocrine tumors in our patient were negative, additional prolactinoma predisposing factors should be at least considered. We propose that a potential role for inactivating variants in the *CASR* gene deserves further study in relation to the pathogenesis of KS. Finally, the long-lasting therapy with sex steroids initiated at adolescence may have facilitated the development of the prolactinomas as similar cases have been reported in the literature. Acknowledging such risk would be important, since sex steroid replacement therapy could mask the hypogonadism as an early sign of prolactinoma.

## 4 Conclusion

We present the case of a young male patient with a rare co-occurrence of KS, multiple prolactinomas and familial hypocalciuric hypercalcemia (FHH). While genetic background of KS and of pituitary adenomas remained unclear, FHH was genetically confirmed by a pathogenic inactivating variant in the *CASR* gene. We are unaware of earlier reports of the combination of these rare clinical features in KS patients and suggest their possible interrelation deserves further attention.

## Data availability statement

The original contributions presented in the study are included in the article/**Supplementary Material**. Further inquiries can be directed to the corresponding author.

## Ethics statement

The case study was conducted according to the guidelines of the Declaration of Helsinki, and approved by the Medical Ethics Committee of the Republic of Slovenia (#29/06/14 and #132/03/15). Written informed consent was obtained from the patient for publication of this case report and any accompanying images.

## Author contributions

MJ: Conceptualization, Data collection, Analysis and Interpretation, Literature review, Writing - original draft, Writing - review and editing; MA: Conceptualization, Data collection, Analysis and Interpretation, Literature review, Writing - original draft, Writing - review and editing and Supervision; TV, MD, NB, KT, and RH: Data collection, Analysis and Interpretation, Writing - review & editing; AJ, EF, and TB: Analysis and Interpretation, Literature review and Writing review & editing. All authors contributed to the article and approved the submitted version.
