# Supplementary material for: Case Report: Multiple prolactinomas in a young man with Kallmann syndrome and familial hypocalciuric hypercalcemia
Source: Front Endocrinol (Lausanne). 2023 Oct 30;14:1248231. doi: 10.3389/fendo.2023.1248231 (PMC10642931; doi:10.3389/fendo.2023.1248231)
Supplement: Supplementary file 1 [file Table_1.docx]

Supplementary Material

Case Report: Multiple prolactinomas in a young man with Kallman syndrome and familial hypocalciuric hypercalcemia

**Mojca Jensterle*, Andrej Janez, Tina Vipotnik Vesnaver, Maruša Debeljak, Nika Breznik, Katarina Trebušak Podkrajšek, Rok Herman, Eric Fliers, Tadej Battelino, and Magdalena Avbelj Stefanija**

*** Correspondence:**Mojca Jensterle
mojcajensterle@yahoo.com

**Suppl Table 1:** Panel of genes associated with neuroendocrine tumors.

| *AIP* | *CDKN1A* | *DICER1* | *MEN1* | *NF1* | *PRKAR1A* | *SDHB* | *SF3B1* | *USP48* |
| --- | --- | --- | --- | --- | --- | --- | --- | --- |
| *ATRX* | *CDKN1B* | *GNAS* | *MLH1* | *NR3C1* | *RET* | *SDHC* | *TP53* | *USP8* |
| *CABLES1* | *CDKN2B* | *GPR101* | *MSH2* | *PMS2* | *SDHA* | *SDHD* | *TSC1* | *VHL* |
| *CDH23* | *CDKN2C* | *MAX* | *MSH6* | *PRKACA* | *SDHAF2* | *SENP8* | *TSC2* | *YY1* |

**Suppl Table 2:** Panel of genes associated with congenital hypogonadotropic hypogonadism.

| *ANOS1* | *DMXL2* | *GNRH1* | *KLB* | *NRP1* | *PLXND1* | *SEMA3A* | *SOX2* |
| --- | --- | --- | --- | --- | --- | --- | --- |
| *AXL* | *FEZF1* | *GNRHR* | *LEP* | *NRP2* | *PNPLA6* | *SEMA3E* | *SOX3* |
| *CCDC141* | *FGF17* | *HESX1* | *LEPR* | *NSMF* | *POLR3B* | *SEMA3F* | *SPRY4* |
| *CHD7* | *FGF8* | *HS6ST1* | *LHB* | *NTN1* | *PROK2* | *SEMA3G* | *TAC3* |
| *CUL4B* | *FGFR1* | *IGSF10* | *LHCGR* | *OTUD4* | *PROKR2* | *SEMA7A* | *TACR3* |
| *DCAF17* | *FLRT3* | *IL17RD* | *LHX3* | *PCSK1* | *PROP1* | *SLC29A3* | *TUBB3* |
| *DCC* | *FSHB* | *KISS1* | *LHX4* | *PLXNA1* | *PTCH1* | *SMCHD1* | *WDR11* |
| *DUSP6* | *GLI2* | *KISS1R* | *NR0B1* | *PLXNA3* | *RNF216* | *SOX10* |  |

**Suppl Table 3:** Panel of genes associated with GnRH neuronal biology (Akram M, Raza Rizvi SS, Qayyum M, Handelsman DJ. A classification of genes involved in normal and delayed male puberty. Asian J Androl. 2022 Apr 29. doi: 10.4103/aja202210. Epub ahead of print)

| *AKAP1* | *CHD4* | *FGF18* | *GRIA3* | *KDR* | *NOTCH1* | *PLXNA3* | *SCEL* | *STS* |
| --- | --- | --- | --- | --- | --- | --- | --- | --- |
| *AKAP2* | *CHD7* | *FGF19* | *GRIA4* | *KISS1* | *NPBWR1* | *PLXNA4* | *SEC14L3* | *STUB1* |
| *AKAP3* | *CLPP* | *FGF2* | *GRIK1* | *KISS1R* | *NPTXR* | *PLXNB1* | *SEC16B* | *STXBP4* |
| *AKAP4* | *CNTN2* | *FGF20* | *GRIK2* | *KLB* | *NPY1R* | *PLXND1* | *SEC23IP* | *SUFU* |
| *ALDH1A1* | *CPE* | *FGF21* | *GRIK3* | *KLF12* | *NR0B1* | *PNPLA6* | *SEMA3A* | *SYCP2* |
| *ALDH1A2* | *CRHR1* | *FGF22* | *GRIK5* | *KMT2D* | *NR3C1* | *POLR1C* | *SEMA3B* | *TAC3* |
| *ALMS1* | *CRTC1* | *FGF3* | *GRIN1* | *LATS1* | *NR5A1* | *POLR3A* | *SEMA3C* | *TACR1* |
| *ALX4* | *CRY1* | *FGF4* | *GRIN2A* | *LEF1* | *NRP1* | *POLR3B* | *SEMA3D* | *TACR2* |
| *AMH* | *CTBP2* | *FGF5* | *GRIN2B* | *LEP* | *NRP2* | *POMC* | *SEMA3E* | *TACR3* |
| *AMHR2* | *CUL4B* | *FGF6* | *GRIN2C* | *LEPR* | *NSMF* | *POU1F1* | *SEMA3F* | *TAX1BP3* |
| *AMN1* | *CXCL12* | *FGF7* | *GRIN2D* | *LGR4* | *NTN1* | *POU3F1* | *SEMA3G* | *TBC1D20* |
| *ANK1* | *CXCR4* | *FGF8* | *GRIN3A* | *LHB* | *NTRK2* | *POU3F2* | *SEMA4D* | *TBCE* |
| *ANOS1* | *CYP19A1* | *FGF9* | *GRIN3B* | *LHCGR* | *NUCKS1* | *POU5F1* | *SEMA7A* | *TBX2* |
| *AR* | *DCAF17* | *FGFR1* | *GRM1* | *LHX1* | *OFD1* | *PRDM13* | *SF1* | *TBX6* |
| *ARL6* | *DCC* | *FGFR1OP2* | *GRM2* | *LHX3* | *OLFM2* | *PROK2* | *SFRP1* | *TCF12* |
| *ARNT2* | *DET1* | *FGFR3* | *GRM3* | *LHX4* | *OLFM3* | *PROKR1* | *SFRP5* | *TCF7* |
| *ARRB2* | *DHH* | *FLRT3* | *GRM5* | *LHX5* | *OPRD1* | *PROKR2* | *SHH* | *TENM2* |
| *ARX* | *DLG2* | *FOXG1* | *GRM7* | *LHX6* | *OPRK1* | *PROP1* | *SIM1* | *TFAP4* |
| *ASCC3* | *DLGAP1* | *FOXH1* | *HAMP* | *MAGEL2* | *OPRM1* | *PTCH1* | *SIM2* | *TFR2* |
| *ASCL1* | *DLK1* | *FRS3* | *HCRTR1* | *MAP1A* | *OR2K2* | *PTCH2* | *SIN3A* | *TGFB1* |
| *AXIN1* | *DLX1* | *FSHB* | *HESX1* | *MAP2K5* | *OTP* | *PTPRD* | *SIX3* | *TLE4* |
| *AXL* | *DLX2* | *FSTL5* | *HFE* | *MAPK14* | *OTUD4* | *PTPRF* | *SIX6* | *TLE5* |
| *B2M* | *DMXL2* | *GAB2* | *HJV* | *MASTL* | *OTX2* | *RAB18* | *SLC29A3* | *TMEM108* |
| *BBS1* | *DNMT3B* | *GABBR1* | *HS6ST1* | *MC4R* | *PALM2* | *RAB3GAP1* | *SLC40A1* | *TMEM38B* |
| *BCL11A* | *DRD1* | *GABRA1* | *HSH2D* | *MCHR2* | *PAX6* | *RAB3GAP2* | *SLIT2* | *TRAPPC9* |
| *BDNF* | *DRD2* | *GALR1* | *HTR1F* | *MEOX1* | *PAX7* | *RARA* | *SMAD5* | *TRIM37* |
| *BEGAIN* | *DUSP6* | *GALR2* | *IFT57* | *MET* | *PCDH7* | *RARB* | *SMCHD1* | *TRPC6* |
| *BEND6* | *EGF* | *GALR3* | *IGF1* | *MKRN3* | *PCDH8* | *RARG* | *SMIM20* | *TSHZ1* |
| *BMP2* | *EGFR* | *GAP43* | *IGF1R* | *NCAM1* | *PCSK1* | *RAX* | *SMO* | *TSPAN11* |
| *BMP4* | *EMX1* | *GDF1* | *IGSF10* | *NDN* | *PDE3A* | *RBM28* | *SOX10* | *TUBB3* |
| *BMP7* | *EMX2* | *GLCE* | *IL17RD* | *NDNF* | *PDGFRB* | *RD3* | *SOX2* | *UBA7* |
| *CA10* | *FAM83B* | *GLI1* | *INSR* | *NEUROD1* | *PEX2* | *RNF144B* | *SOX3* | *VAX1* |
| *CACNA1B* | *FEZF1* | *GLI2* | *IRF2BPL* | *NEUROG2* | *PGM1* | *RNF216* | *SP8* | *WDR11* |
| *CADM2* | *FGF1* | *GLI3* | *IRX3* | *NEUROG3* | *PIN1* | *ROBO1* | *SPARCL1* | *WDR4* |
| *CCDC141* | *FGF10* | *GNAS* | *IRX5* | *NHLH1* | *PKNOX1* | *ROBO2* | *SPRED3* | *WNT11* |
| *CCDC28B* | *FGF11* | *GNRH1* | *JADE2* | *NHLH2* | *PKNOX2* | *RORA* | *SPRY4* | *WNT8B* |
| *CCDC88C* | *FGF12* | *GNRHR* | *JAG1* | *NKX2-1* | *PLCL1* | *RORB* | *SPX* | *WWP2* |
| *CCKAR* | *FGF13* | *GPRC5B* | *KCNK9* | *NKX2-2* | *PLEKHA5* | *RORC* | *SRA1* | *ZNF131* |
| *CCKBR* | *FGF14* | *GPX6* | *KCTD11* | *NKX6-2* | *PLPP4* | *RXRA* | *STIL* |  |
| *CDKN1C* | *FGF16* | *GRIA1* | *KCTD13* | *NODAL* | *PLXNA1* | *RXRB* | *STK38L* |  |
| *CDON* | *FGF17* | *GRIA2* | *KDM6A* | *NOS1* | *PLXNA2* | *RXRG* | *STMN1* |  |

**Suppl Table 4:** Panel of genes downregulated in prolactinoma cells (Ghatnatti V, Vastrad B, Patil S, Vastrad C, Kotturshetti I. Identification of potential and novel target genes in pituitary prolactinoma by bioinformatics analysis. AIMS Neurosci. 2021 Feb;8(2):254-283)

| *ADAMTSL1* | *CCL3L3* | *DCAF12L2* | *GJA4* | *KIF11* | *NEK2* | *POU3F3* | *RNF215* | *TRHDE* |
| --- | --- | --- | --- | --- | --- | --- | --- | --- |
| *ADD2* | *CCNA1* | *DCAF4L2* | *GLYATL3* | *KIF14* | *NLGN1* | *POU3F4* | *RUFY4* | *TRIM24* |
| *AKAP3* | *CCNB2* | *DEFA3* | *GOLGA6A* | *KIF15* | *NPVF* | *PPBP* | *RYR2* | *TROAP* |
| *AMIGO2* | *CCNF* | *DHX15* | *GPR161* | *KIF18A* | *NRG1* | *PPP4R4* | *SCEL* | *TTK* |
| *ANKFY1* | *CCNG2* | *DISC1* | *GPR17* | *KIF20A* | *NUF2* | *PRC1* | *SEL1L* | *UNC13A* |
| *ANTXR2* | *CDCA5* | *DLEU1* | *GPR26* | *KIF21B* | *NUSAP1* | *PRR11* | *SERINC2* | *UNC5D* |
| *ARHGAP20* | *CDKN3* | *DLGAP5* | *GPR6* | *KIF23* | *NXPH4* | *PSG11* | *SLC19A1* | *VWA1* |
| *ARHGAP29* | *CELA3A* | *DOK6* | *GRIK2* | *KIF26A* | *OMP* | *PSG5* | *SLC25A15* | *VWF* |
| *ASPDH* | *CENPA* | *EPHB1* | *GRIP1* | *KIR3DX1* | *OR6T1* | *PSTPIP2* | *SLC39A5* | *XKR6* |
| *ASXL2* | *CENPH* | *EXD1* | *GRM5* | *KLHL13* | *OXR1* | *PTP4A1* | *SLITRK6* | *YTHDF1* |
| *ATP2A3* | *CENPM* | *EYS* | *GTSE1* | *KRT28* | *PARD6G* | *PTTG2* | *SOX4* | *ZC3H12D* |
| *ATP2B2* | *CHRNA7* | *FAM20C* | *HAUS6* | *L1CAM* | *PAX4* | *QSOX1* | *SPC24* | *ZMAT3* |
| *B4GALT6* | *CKS2* | *FAM71D* | *HIGD1B* | *LCE1B* | *PBK* | *RACGAP1* | *SPC25* | *ZNF160* |
| *BDNF* | *CLTC* | *FAM72D* | *HJURP* | *LRFN1* | *PF4* | *RAD21L1* | *SPEF2* | *ZSCAN12* |
| *BIRC5* | *CNKSR2* | *FAM83A* | *HMGB4* | *LYNX1* | *PKLR* | *RAD51* | *SPINK5* | *ZSCAN2* |
| *BUB1* | *COLEC10* | *FAM91A1* | *HMMR* | *MAGEB2* | *PLA2G2D* | *RASSF10* | *STMN1* |  |
| *C1orf189* | *CPN1* | *FANCA* | *HS3ST2* | *MELK* | *PLCD4* | *RASSF3* | *SV2C* |  |
| *C20orf203* | *CTAG1A* | *FANCD2* | *HSD3B2* | *MND1* | *PLK1* | *RBPJL* | *TAS2R7* |  |
| *C3orf52* | *CXCR2* | *FBXL5* | *HTR3D* | *MTBP* | *PNPLA7* | *RCC1* | *TLE4* |  |
| *CADPS2* | *CXCR3* | *FIBCD1* | *IGFL3* | *MYBPC1* | *POC1A* | *RFC3* | *TMEM40* |  |
| *CALHM1* | *DAB1* | *FOXM1* | *INHBE* | *MYO1D* | *POLQ* | *RFT1* | *TOP2A* |  |
| *CASK* | *DAZAP2* | *G2E3* | *KIAA1958* | *NCAPG* | *POU3F2* | *RGS5* | *TPX2* |  |

**Suppl. Table 5:** Selected variants of unknown significance identified in the proband:

| Gene | Gene variant | Amino acid change | Rs# | GnomAD frequency* | *In silico* predictions† | CADD score§ | ACMG criteria¶ | Family segregation |
| --- | --- | --- | --- | --- | --- | --- | --- | --- |
| *POLR3B* | c.491G>A NM_001160708.2 | p.R164Q | / | <0.01% | uncertain | 24 | PM2. PP2 | Father, sister |
| *PLXNA2* | c.4360G>T NM_025179.4 | p.A1454S | rs767473837 | <0.01% | uncertain | 33 | PM2 | Father, sister |
| *PAX6* | c.800A>C NM_001368929.2 | p.K267T | rs572377074 | <0.01% | uncertain | / | PM2, PP2 | Father |
| *ROBO1* | c.2651G>A NM_001145845 | p.R884Q | rs773504767 | <0.01% | uncertain | 24.7 | PM2 | Mother |
| *PLXNA4* | c.643G>A NM_020911 | p.A215T | rs142904267 | 0.326% | uncertain | 23.5 | PM2, PP2 | Mother, sister |
| *TRAPPC9* | c.2927T>C NM_031466 | p.L967P | rs769348408 | <0.01% | uncertain | 23.9 | PM2, BP4 | Father |

*https://gnomad.broadinstitute.org/ (accessed 6th Dec 2022)

†https://franklin.genoox.com/clinical-db/home (accessed 6th Dec 2022)

§https://cadd.gs.washington.edu/snv (accessed 6th Dec 2022)

* Richards S, Aziz N, Bale S, Bick D, Das S, Gastier-Foster J, et al; ACMG Laboratory Quality Assurance Committee. Standards and guidelines for the interpretation of sequence variants: a joint consensus recommendation of the American College of Medical Genetics and Genomics and the Association for Molecular Pathology. Genet Med. 2015 May;17(5):405-24.
